# Efficient plant regeneration via protocorm-like body induction from stem nodes in *Anoectochilus roxburghii* (Wall.) Lindl.

**DOI:** 10.1186/s12870-026-09210-5

**Published:** 2026-06-06

**Authors:** Shang-yan Fu, Jing-rong Gao, Heng-yu Huang, Yuan-zhong Wang

**Affiliations:** 1 Yunnan Breeding and Research and Development Center of Endangered and Daodi Chinese Medicinal Materials, Yunnan University of Chinese Medicine, Yuhua Road, Chenggong District, Kunming, 650500 China; 2 Medicinal Plants Research Institute, Yunnan Academy of Agricultural Sciences, Kunming, 650200 China

**Keywords:** Endangered medicinal plant, Genetic stability, Plant growth regulators (PGRs), Stem segment explants

## Abstract

*Anoectochilus roxburghii* (Wall.) Lindl., a highly valued yet endangered medicinal orchid, faces significant challenges in propagation due to its limited natural regeneration capacity. This study established an efficient in vitro regeneration system to achieve rapid large-scale propagation, with clear culture cycles for each stage: stem segment explants were subjected to 110 d of initiation culture to obtain sterile materials, 160 d of protocorm-like body (PLB) induction culture to achieve a high proliferation coefficient, 90 d of rooting culture, and 60 d of acclimatization and transplantation. Stem segment explants with nodes were used as explants and cultured on an initiation medium. Through combined full-factorial and orthogonal experiments, the optimal hormone combinations for different regeneration pathways were identified. Notably, a high frequency of PLBs was directly induced from axillary bud meristems without an intervening callus phase on the medium supplemented with 1.5 mg/L 6-BA, 1.0 mg/L NAA, and 0.1 mg/L KT, a regeneration mode that is rare in *A. roxburghii*, achieving a remarkably high proliferation coefficient of over 30.0. Furthermore, the optimal rooting medium was full-strength Murashige and Skoog (MS) medium supplemented with 0.5 mg/L NAA, yielding a 100% rooting rate. The rooted plantlets were successfully acclimatized with a survival rate exceeding 95%. Flow cytometry analysis showed no ploidy variation in regenerated materials at different culture stages, confirming the genetic stability of the system. The findings highlight a novel and efficient PLB-mediated regeneration pathway, which provides a robust technical foundation for the conservation and commercial production of this threatened medicinal plant.

## Introduction

The genus *Anoectochilus* (Orchidaceae) comprises perennial terrestrial herbs, with approximately 60 species distributed mainly in tropical and subtropical regions of Asia. China is home to 20 species, among which *A. roxburghii* has the widest distribution and the most extensive application [1, 2]. It is commonly known as Jinxianlan, Jincan, Jinshisong, Shucaolian, Niaorenshen, Jinxianhutoujiao, or Jinxianruguxiao, with its common Chinese names (or local names) being Jinxianlan and Huayekaichunlan. *A. roxburghii*, along with *A. formosanus* Hayata and *A. chapaensis* Gagnep., serves as the original plant for the traditional Chinese medicine “Jinxianlian“ [3]. *A. roxburghii* possesses creeping, jointed fleshy roots, with leaves that are black-purple or dark purple adaxially, featuring glossy golden-red reticulate veins, giving it high ornamental value. The whole plant is used medicinally; it is sweet in taste and neutral in nature, with effects of clearing heat, cooling blood, eliminating dampness, and detoxifying. It is used to treat tuberculosis with hemoptysis, diabetes, nephritis, cystitis, myasthenia gravis, nocturnal emission, rheumatoid arthritis and osteoarthritis, infantile convulsion, leukorrhea, and snake bites [3, 4]. Pharmacological studies have shown that *A. roxburghii* contains active components such as alkaloids, saponins, and polysaccharides, which exhibit good therapeutic effects against cancer, hyperlipidemia, diabetes, inflammation, and liver injury [5, 6]. Its unique leaf structure, distinct vein coloration, and significant bioactivity confer high commercial value to *A. roxburghii*. It is listed in Appendix II of the Convention on International Trade in Endangered Species of Wild Fauna and Flora (CITES) and classified as a National Grade II Protected Plant in China [4].

Due to substantial market demand and ongoing habitat destruction, wild populations of *A. roxburghi* have become extremely rare. To realize the sustainable utilization of its high commercial value, urgent research on the artificial propagation of *A. roxburghii* is required. Seed propagation is a common regeneration method for many plants, but it is difficult to achieve for *A. roxburghii*. Similar to most orchids, the seeds of *A. roxburghii* are minute and lack endosperm; their germination depends on mycorrhizal fungi for nutrition until the seedlings develop green leaves and become autotrophic [2]. Consequently, under natural or semi-natural field conditions, seed propagation faces problems such as a long germination cycle, low germination rate, and low seedling establishment rate. Although tillering propagation can maintain parental traits, each rhizome produces only 1–2 buds, which also presents limitations of low efficiency and a long cycle, making it difficult to meet the demands of industrial production [7]. Since Knudson established the technique of aseptic asymbiotic germination in 1922, the large-scale production of many high-value orchids has become possible [8]. Within micropropagation systems aimed at obtaining robust seedlings, various morphogenetic pathways have been developed, among which the formation of protocorm-like bodies (PLBs) and protocorms significantly improves the propagation efficiency of orchids. PLBs typically refer to embryogenic tissues morphologically similar to protocorms, formed in vitro from explants other than seeds; these structures, derived from explants or callus, are considered somatic embryos with high regenerative potential [9, 10]. Tissue culture technology remains an important method for the large-scale clonal propagation of superior hybrids and commercial varieties of orchids due to its high multiplication rate, short cycle, and operational convenience. Therefore, the induction and formation mechanisms of protocorms and protocorm-like bodies remain a key research focus in orchidology.

Existing studies have shown that seeds, shoot tips, stem segments, and leaves of *A. roxburghii* can achieve in vitro regeneration, but the efficiency remains unsatisfactory [11]. At present, the reported PLB induction systems for A. roxburghii mostly take callus as the intermediate receptor, which has the problems of low proliferation efficiency, long culture cycle and high risk of somaclonal variation [12, 13]. Some studies have also induced PLBs from stem segments and indicated that dark or low-light conditions significantly affect their proliferation and differentiation; however, the proliferation efficiency and growth cycle still fall short of ideal levels [14]. This study used stem segments explants of *A. roxburghii* as explants with the aim of inducing axillary bud regeneration while simultaneously investigating the physiological and morphological mechanisms of PLB formation directly from the stem nodes. The goal was to establish a stable pathway for PLBs formation and thereby construct a highly efficient plant regeneration system, which effectively avoids the technical defects of existing systems. This research is expected to provide technical support for cultivating genetically stable, high-quality *A. roxburghii* seedlings, further enhance artificial propagation efficiency, and thereby alleviate the challenges in demand and quality faced by the seedling market at the source. It also offers a reference for the efficient in vitro propagation of other orchid species.

## Materials and methods

### Terminology definition

Axillary bud: Directly derived from the meristem of stem segment explants, the initial bud structure differentiated from the node of *A. roxburghii*.

Adventitious bud: Secondarily differentiated from PLBs, with the potential to develop into complete plantlets.

Multiplication coefficient: The number of robust axillary buds formed per explant, used for the adventitious bud induction stage.

Proliferation coefficient: The total number of robust axillary buds + PLBs formed per explant (PLBs are counted as effective individuals that can differentiate into seedlings), used for the PLB induction stage.

### Plant materials

The experimental materials consisted of 20 potted plants, totaling 60 robust *A. roxburghii* individuals, kindly provided by Dr. Ding Changchun from Wenshan University, Yunnan, China. The plants (still in their original pots) were transplanted to the experimental greenhouse of the Engineering Research Center for High-Quality Seed and Seedling Breeding of Chinese Medicinal Materials, Yunnan University of Chinese Medicine (Geographical coordinates: 25°24’36’’N, 103°22’10’’E; Altitude: 1878.3 m). All materials were identified as *Anoectochilus roxburghii* (Wall.) Lindl. by Professor Heng-yu Huang of Yunnan University of Chinese Medicine. A voucher specimen has been deposited in the Herbarium of the Medicinal Plant Research Institute, Yunnan Academy of Agricultural Sciences, China, with the specimen number HHY-2023-065.

### Preparation and initiation culture of explants

Stem segments explants with 3–5 nodes (leaves removed) were rinsed under running water for 30 min and then placed in a laminar flow hood. They were sequentially sterilized with 75% (v/v) ethanol for 10 s, followed by treatment with a 0.1% (w/v) aqueous mercuric chloride (HgCl₂) solution (with gentle shaking) for 6–10 min, and finally rinsed three times with sterile water, each rinse lasting no less than 3 min. The sterilized stem segments explants were placed on sterile absorbent paper to remove surface moisture, and then cut into small sections approximately 2–3 cm in length, each containing 1–2 nodes, using a sterile scalpel. These sections were vertically inserted into the initiation medium in accordance with their physiological orientation.

### Basal medium

Murashige and Skoog (MS) medium (1962) [15] was used in all culture stages of this study, including the initiation culture stage. The initiation medium consisted of MS basal medium supplemented with 1.0 mg/L 6-benzyladenine (6-BA), 0.1 mg/L α-naphthalene acetic acid (NAA), 1 g/L activated charcoal, 30 g/L banana pulp homogenate, 30 g/L apple pulp homogenate, 30 g/L sucrose, and 4.7 g/L agar, with the pH adjusted to 5.4–5.8 [16, 17]. This formulation was based on established orchid culture systems previously optimized in our laboratory. Growth and development of the explants were regularly observed and recorded every 30 d during the 110 d initiation culture period.

The plant growth regulators (PGRs) employed in other culture stages, including 1-naphthaleneacetic acid (NAA), 2,4-dichlorophenoxyacetic acid (2,4-D), kinetin (KT, IUPAC name: 6-furfurylaminopurine), and 6-benzylaminopurine (6-BA; chemical name: N-(phenylmethyl)-9 H-purin-6-amine), as well as sucrose and agar, were of analytical grade and purchased from Beijing Dingguo Biotechnology Co., Ltd. Unless otherwise specified, all hormone concentrations are expressed as mass concentrations. The MS medium was supplemented with 30 g/L sucrose and 4.7 g/L agar, dissolved in deionized water, and the pH was adjusted to 5.6–5.8. The medium was sterilized by autoclaving at 122 °C (103 kPa) for 22 min before use.

### Screening of PGR types and concentration ranges

MS basal medium was supplemented with different concentrations of individual plant growth regulators (PGRs): 6-BA (1.0, 1.5, 2.0, 2.5, 3.0 mg/L), KT (0.1, 0.5, 1.0, 1.5, 2.0 mg/L), NAA (0.1, 0.5, 1.0, 1.5, 2.0 mg/L), and 2,4-D (0.01, 0.05, 0.1, 0.5, 1.0 mg/L). Stem segment and shoot tip explants were inoculated into the media supplemented with each regulator at the specified concentrations (i.e., single-hormone treatments). After 90 d of culture, the growth status (e.g., explant browning, elongation, and callus formation) and adventitious bud induction efficiency of the materials in each treatment group were recorded and analyzed to screen suitable types and concentration ranges of PGRs for subsequent experiments.

Each treatment was replicated three times. For stem segment explants, each replicate consisted of 5 bottles, with 8 explants per bottle (total 120 explants per treatment). Due to the limited availability of shoot tip materials, each replicate of the shoot tip treatment group was inoculated into 1 bottle (8 explants per bottle), with three replicates in total (total 24 explants per treatment).

### Experiments with different concentration combinations of PGRs

Based on the results of the plant growth regulator (PGR) screening experiment, 6-BA (1.0, 1.5, 2.0 mg/L) and NAA (0.5, 1.0, 1.5 mg/L) were selected for a full factorial combination experiment, resulting in 9 treatment groups (3 concentrations of 6-BA × 3 concentrations of NAA). This experiment aimed to evaluate the effects of different concentration ratios of these two PGRs on adventitious bud induction from stem segment explants. After 90 d of culture, the multiplication coefficient of each combination was calculated. Each treatment was replicated three times, with each replicate consisting of 10 bottles and each bottle inoculated with 10 stem segment explants (total 300 stem segment explants per treatment).

### Propagation experiment

Based on the results of the PGR screening and combination experiments, three key factors—6-BA (Factor A), NAA (Factor B), and KT (Factor C)—were selected and arranged according to an L_9_(3⁴) orthogonal design. The L_9_ (3^4^) orthogonal design was selected because this study needs to investigate 3 factors (3 levels each) including 6-BA, NAA and KT; this design can realize the comprehensive combination of factors and levels through a minimum of 9 experiments, which is both efficient and scientific. The design included 9 treatments, 3 levels per factor, 4 columns; 3 columns for the three factors, 1 column as a blank for error analysis. The blank column (4th column) was used to calculate the experimental error, and the significance of the effect of each factor on PLB proliferation was verified by comparing the range of the factor column with the blank column; the range of the blank column (0.38) was much lower than that of each factor, indicating small experimental error and reliable results. The level combinations of each factor are presented in Table 1.

**Table 1:** Orthogonal design L_9_ (3^4^) for the proliferation of *A. roxburghii*

Levels		Factors ( mg·L^− 1^)	
	A (6-BA)	B (NAA)	C (KT)
1	1.0	0.1	0.1
2	1.5	0.5	0.5
3	2.0	1.0	1.0

Stem segment explants were inoculated into the respective treatment media, and after 90 d of culture, the growth status of explants was observed, and the proliferation coefficient was calculated. Each treatment was replicated three times, with each replicate consisting of 10 bottles and each bottle inoculated with 10 stem segment explants.

### Culture conditions

The culture room was maintained at a temperature of (22 ± 1)°C, with a light intensity of 1500–2000 lx (provided by cool-white fluorescent lamps) and a photoperiod of 10 h/d. Contaminated cultures were promptly discarded, and experimental culture bottles were replenished with fresh explants to maintain the required sample size for each treatment group.

### Flow cytometry analysis

Fresh leaf tissues(0.5 g per sample) from materials at different cultivation stages (A11 = primary cultured seedlings, A12 = PLB proliferation stage seedlings, A13 = rooting cultured seedlings) were vertically minced and left undisturbed in LB01 dissociation solution (15 mmol/L Tris, 2 mmol/L Na₂EDTA, 0.5 mmol/L spermine tetrahydrochloride, 80 mmol/L KCl, 20 mmol/L NaCl, 0.1% (v/v) Triton X-100, and 15 mmol/L β-mercaptoethanol, adjusted to pH 7.0–8.0. The storage conditions (stored at -20 °C and thawed before use, then kept at 4 °C after thawing), Coolaber, Beijing, China) for 10 min. The cell nuclei suspension was obtained by filtering the mixture. For PI staining, PI staining solution (50 µg/mL, containing RNase A, Coolaber, Beijing, China) was added to the suspension, followed by incubation on ice under light-shielded conditions for 0.5–1 h. The samples were then analyzed using a CytoFLEX flow cytometer (Beckman Coulter, Suzhou, China). The 488 nm blue light excitation was employed to detect the emission fluorescence intensity of PI, with 10,000 cells collected per run. The coefficient of variation (CV%) was maintained below 5%. The experiment was set with 3 biological replicates, and no technical replicates were performed. Due to the lack of an appropriate internal reference with a known genome size in the laboratory, no internal standard was included in the analysis. Graphical analysis was performed using Modifit3.0 software [18].

### Rooting and acclimatization transplantation experiment

Based on the results of the PGR screening experiment, NAA (0, 0.1, 0.5, 1.0 mg/L) was added to the full-strength MS basal medium for rooting culture. Regenerated shoots with a height of 3–5 cm were inoculated into the respective treatment media. After 90 d of culture, the rooting rate and the average number of adventitious roots per seedling were recorded. Each treatment was replicated three times, with each replicate consisting of 10 bottles and each bottle inoculated with 10 explants.

After 90 d of rooting culture, the seedlings grew robustly with thick root systems and reached a height of 5–7 cm.

The cultivation containers used were foam boxes with internal dimensions of 13 cm × 18 cm × 15 cm, and the humus soil substrate was filled to a thickness of 6 cm. The culture bottles were first placed under natural light (2000–3000 lx) for acclimatization for 5 ds, after which the sealing film was removed for an additional 2 d of acclimatization. Humidity was controlled by gradually uncovering the sealing film: the 1/2 sealing film was retained to maintain 80% relative humidity for 3 d, and then the sealing film was completely uncovered to maintain 70% relative humidity for 2 d.The seedlings were gently removed from the bottles, and the medium attached to the roots was carefully washed off with sterile water. They were then soaked in a 0.1% (w/v) carbendazim (methyl benzimidazol-2-ylcarbamate) solution for 5 min and transplanted into high-temperature-sterilized humus soil. Mycorrhizal fungi were not inoculated in this study, and the high-temperature sterilized humus soil in the acclimatization medium could meet the adaptation needs of the seedlings, with a transplant survival rate of more than 95%.

The transplanted seedlings were cultivated under conditions of 20–25 °C and approximately 70% relative humidity for 60 ds, after which the transplantation survival rate was recorded (seedlings with new leaf growth and turgid leaves were considered survived).

### Data processing

The results of all treatment groups are expressed as mean ± standard error (SE). One-way analysis of variance (ANOVA) was performed using SPSS 26.0 software, and significant differences between groups were compared using Duncan’s multiple range test at a significance level of α = 0.05. The calculation formulas for each indicator are arranged according to the experimental workflow as follows:

$$\begin{aligned} &\mathrm{Average}\;\mathrm{number}\;\mathrm{of}\;\mathrm{adventitious}\;\mathrm{roots}\;\mathrm{per}\;\mathrm{plantlet}\;\\&=\;\mathrm{Total}\;\mathrm{number}\;\mathrm{of}\;\mathrm{adventitious}\;\mathrm{roots}\\&\;/\;\mathrm{Total}\;\mathrm{number}\;\mathrm{of}\;\mathrm{explants}\;\mathrm{that}\;\mathrm{formed}\;\mathrm{adventitious}\;\mathrm{roots} \end{aligned}$$

$$\begin{aligned} &\mathrm{Axillary}\;\mathrm{bud}\;\mathrm{induction}\;\mathrm{rate}\;(\%)\;\\&=\;(\mathrm{Number}\;\mathrm{of}\;\mathrm{axillary}\;\mathrm{bud}-\mathrm{producing}\;\mathrm{explants}\\&\;/\;\mathrm{Total}\;\mathrm{number}\;\mathrm{of}\;\mathrm{initially}\;\mathrm{inoculated}\;\mathrm{explants})\;\times\;100 \end{aligned}$$

$$\begin{aligned} &\mathrm{Proliferation}\;\mathrm{coefficient}\;\\&=\;\mathrm{Number}\;\mathrm{of}\;\mathrm{effective}\;\mathrm{subcultured}\;\mathrm{explants}\;\\&(\mathrm{defined}\;\mathrm{as}\;\mathrm{robust}\;\mathrm{adventitious}\;\mathrm{buds}\\&/\mathrm{PLBs}\;\mathrm{suitable}\;\mathrm{for}\;\mathrm{subculture},\;\mathrm{with}\;\text a\;\mathrm{length}\;\geq\;1\;\mathrm{cm})\;\\&/\;\mathrm{Number}\;\mathrm{of}\;\mathrm{initially}\;\mathrm{inoculated}\;\mathrm{explants} \end{aligned}$$

$$\begin{aligned} &\mathrm{Protocorm}-\mathrm{like}\;\mathrm{body}\;(\mathrm{PLB})\;\mathrm{induction}\;\mathrm{rate}\;(\%)\;\\&=\;(\mathrm{Number}\;\mathrm{of}\;\mathrm{explants}\;\mathrm{with}\;\mathrm{node}-\mathrm{derived}\;\mathrm{PLBs}\;\\&/\;\mathrm{Total}\;\mathrm{number}\;\mathrm{of}\;\mathrm{initially}\;\mathrm{inoculated}\;\mathrm{explants})\;\times\;100 \end{aligned}$$

$$\begin{aligned} &\mathrm{Rooting}\;\mathrm{rate}\;(\%)\;\\&=\;(\mathrm{Number}\;\mathrm{of}\;\mathrm{explants}\;\mathrm{forming}\;\mathrm{adventitious}\;\mathrm{roots}\;\\&/\;\mathrm{Total}\;\mathrm{number}\;\mathrm{of}\;\mathrm{initially}\;\mathrm{inoculated}\;\mathrm{explants})\;\times\;100 \end{aligned}$$

$$\begin{aligned} &\mathrm{Transplantation}\;\mathrm{survival}\;\mathrm{rate}\;(\%)\;\\&=\;(\mathrm{Number}\;\mathrm{of}\;\mathrm{surviving}\;\mathrm{plantlets}\;\\&/\;\mathrm{Total}\;\mathrm{number}\;\mathrm{of}\;\mathrm{transplanted}\;\mathrm{seedlings})\;\times\;100 \end{aligned}$$

## Results and analysis

### Preliminary propagation of sterile materials

**Fig. 1:**
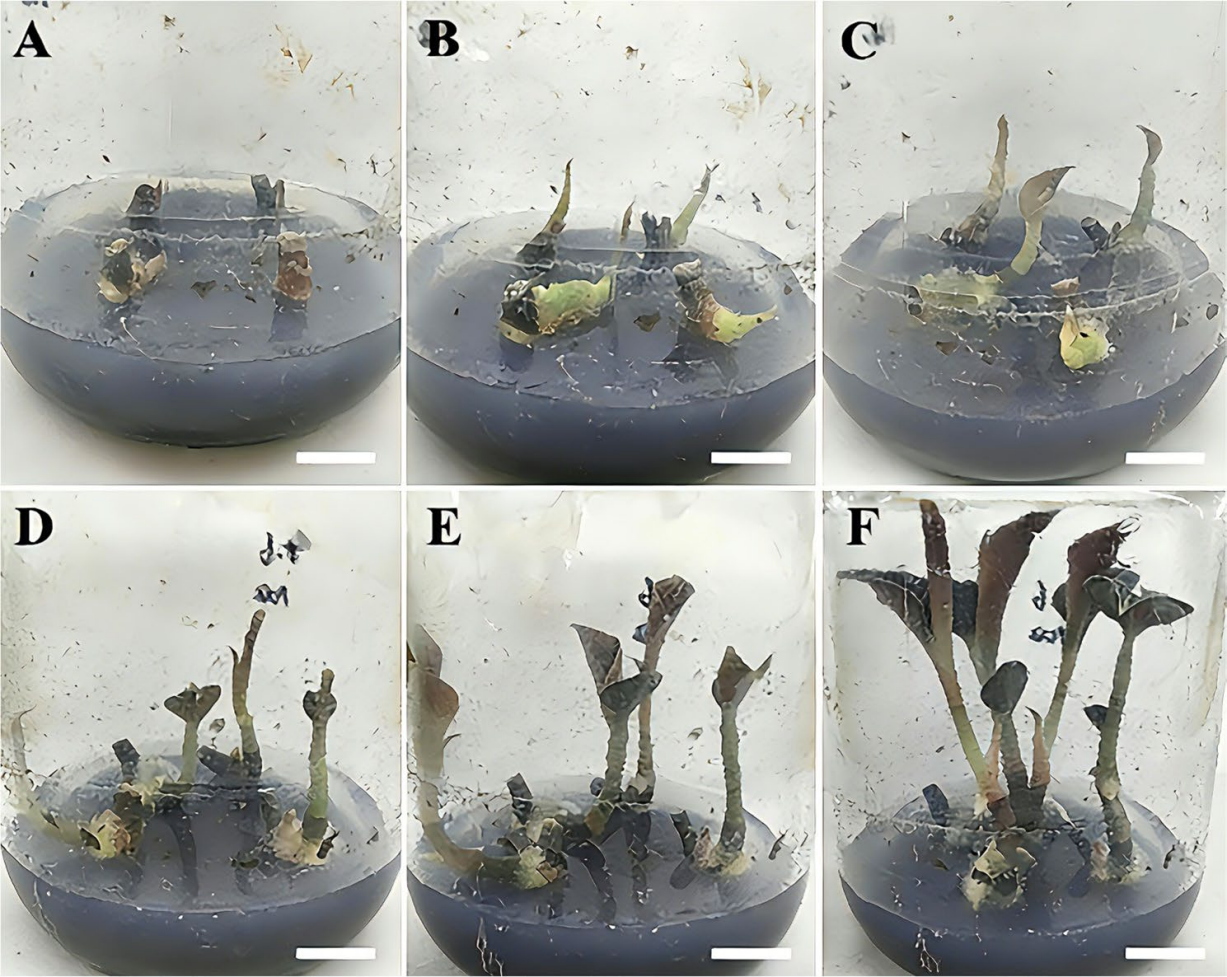
Acquisition and initial culture of sterile materials of *A. roxburghii*. **A** Stem segment explants inoculated in the initial basal medium after sterilization; **B** Growth status after 30 d of culture; **C** Growth status after 60 d of culture; **D** Growth status after 90 d of culture; **E** Growth status after 110 d of culture; **F** Growth status after 130 d of culture. (Scale = 2 cm)

Sterilized stem segment explants were inoculated into the initial basal medium (Fig. 1A). After 30 d of culture, green buds emerged from the nodes, with white filamentous structures (presumed to be early-stage protocorm-like structures, PLBs) observed at the base of the buds (Fig. 1B). After 60 d of culture, young leaves began to unfold at the apex of the buds (Fig. 1C). After 90 d of culture, the buds continued to elongate, with the second leaf gradually developing and the number of filamentous structures at the base further increasing (Fig. 1D). After 110 d of culture, the number of leaves at the bud apex significantly increased (Fig. 1E). After 130 d of culture, the leaves were fully expanded, with distinct stem nodes and densely arranged ring-like filamentous structures at the internodes of the base (Fig. 1F). Stable propagation of sterile materials was achieved through 3–5 subcultures in this medium, with a subculture interval of 90 ds.

### Effects of different types and concentrations of PGRs on the growth of *A. roxburghii* (Wall.) Lindl.

The results indicated that different PGRs significantly influenced the growth and development of *A. roxburghii*. High concentrations of cytokinins (6-BA and KT) induced node swelling and inhibited normal stem elongation, while auxins (NAA and 2,4-D) played a key role in adventitious root induction.

**Fig. 2:**
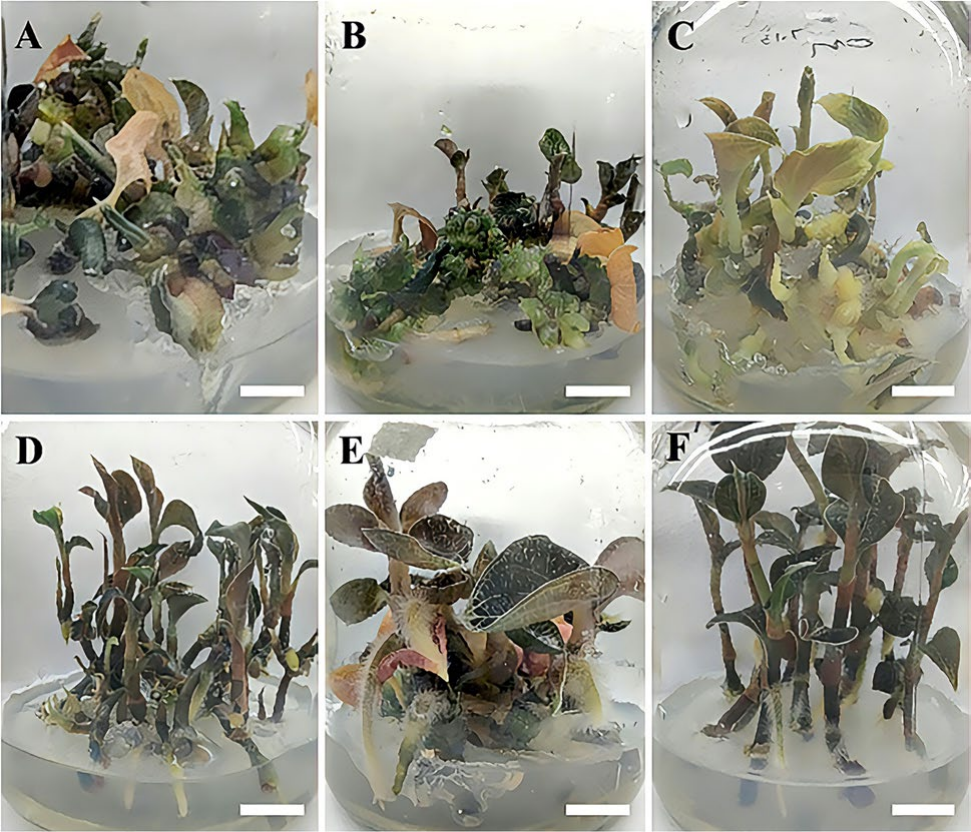
Growth results of stem segments and shoot tip explants of *A. roxburghii* in different types and concentrations of PGRs. **A** Adventitious bud clumps occurrence in MS medium with 1.5 mg·L^− 1^ 6-BA; **B** Abnormal adventitious bud clumps occurrence in MS medium with 2.5 mg·L^− 1^ 6-BA; **C** Plants were deformed and yellowed, and occasionally adventitious buds were found in MS medium with 1.5 mg·L^− 1^ KT; **D** Adventitious roots and axillary buds were found in MS medium with 0.5 mg·L^− 1^ NAA; **E** In MS medium with 0.5 mg·L^− 1^ 2,4-D, the plant grew slowly, the base swelled in volume and adventitious roots were found; **F** Shoot tips grew rapidly and adventitious roots were obvious in MS medium with 0.5 mg·L^− 1^ NAA. Scale bar = 2.0 cm. Note: Each group was repeated with 10 bottles and 10 seedlings per bottle

When the concentration of 6-BA was 1.5 mg/L, green adventitious bud clusters formed at the nodes, accompanied by significantly inhibited stem growth (Fig. 2A). At 6-BA concentrations of 2.5–3.0 mg/L, the nodes became markedly swollen, forming irregular emerald-green callus masses with occasional malformed adventitious buds, and stem growth was severely inhibited or even led to death (Fig. 2B). In KT treatments, adventitious buds occasionally emerged at the nodes. When the KT concentration reached 1.5 mg/L, stem growth slowed, the base swelled, and leaves became deformed and chlorotic (Fig. 2C). NAA treatments consistently induced adventitious root formation, with roots being relatively robust within the range of 0.1–1.5 mg/L. At concentrations of 0.5–1.0 mg/L, each stem segment explant produced 1–2 axillary buds (Fig. 2D). 2,4-D at concentrations of 0.01–0.1 mg/L supported normal plant growth (e.g., upright stems, uniformly green leaves) and induced adventitious roots. However, when the concentration increased to 0.5 mg/L, although adventitious roots were induced, the stem base swelled, and growth was significantly inhibited (Fig. 2E).

Additionally, across all treatments, adventitious buds were not induced from shoot tips; only NAA treatment induced adventitious root formation from shoot tips, suggesting that shoot tips of *A. roxburghii* are more responsive to auxins for root induction than for bud induction (Fig. 2F).

### Effects of different concentration combinations of PGRs on the regeneration of *A. roxburghii* (Wall.) Lindl.

**Fig. 3:**
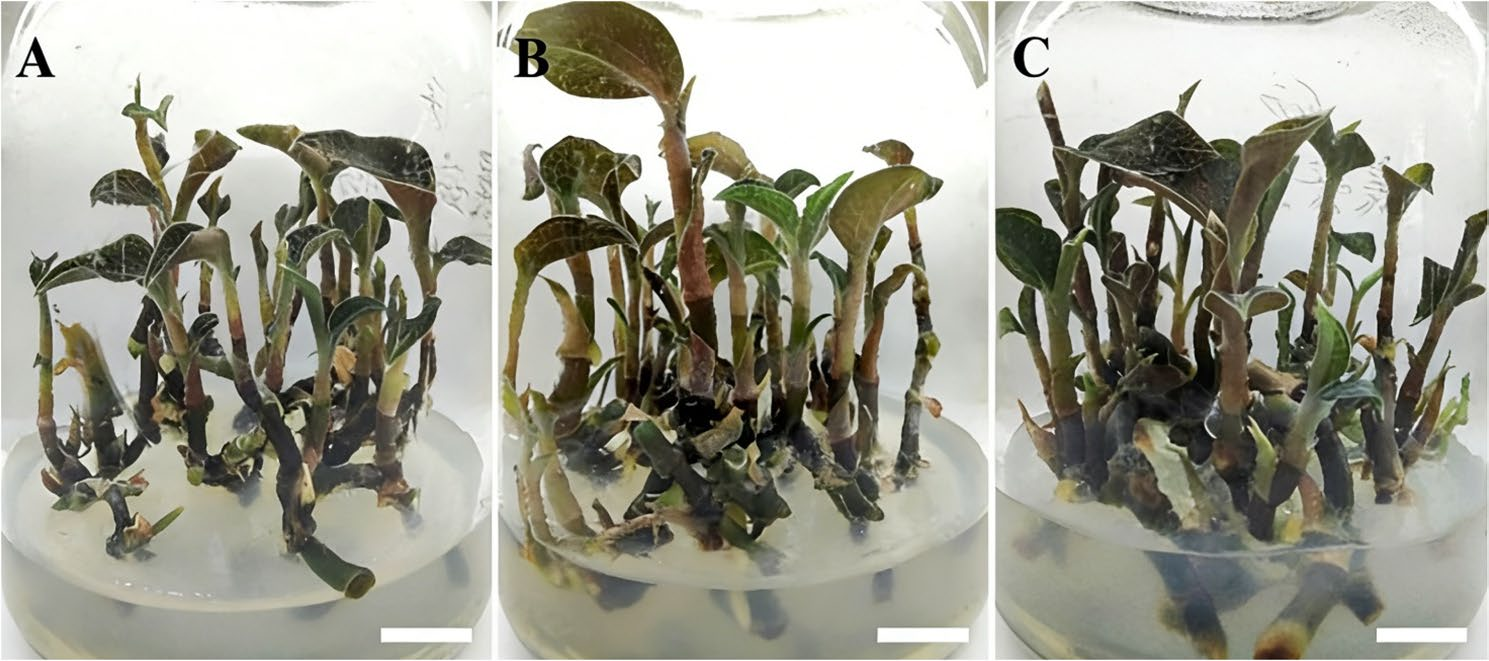
Result of different combinations of PGRs and subculture *A. roxburghii*. **A** After 90 d of culture, 2 ~ 3 axillary buds occurred in each stem segment explant in MS medium with 2.0 mg·L^− 1^ 6-BA and 0.5 mg·L^− 1^ NAA; **B** After the first subculture of the same stem segment explant for 90 ds, the stem segment swelled in volume and axillary buds increased; **C** After the second subculture of the same stem segment explant for 90 ds, the stem segment continued to swell and produced 4–5 axillary buds on average. (Scale bar = 2.0 cm)

The results of the full factorial combination experiment are shown in Table 2. When the concentration of 6-BA was fixed, the proliferation coefficient initially increased and then decreased with rising NAA concentrations. At lower NAA concentrations (< 1.0 mg/L), the proliferation coefficient improved with increasing 6-BA levels. The combination of high 6-BA and low NAA concentrations promoted the differentiation of multiple axillary buds at the stem nodes of explants. Among all treatments, the medium supplemented with 2.0 mg/L 6-BA and 0.5 mg/L NAA was most conducive to axillary bud proliferation, with each stem segment explant producing an average of 2–3 axillary buds and a multiplication coefficient of approximately 4.0 (the highest among all combinations; Table 2; Fig. 3A). 

**Table 2:** Effects of different combinations of plant growth regulators on regeneration of *A. roxburghii*

NO.	PGRs(mg·L^− 1^)		Multiplication coefficient
	6-BA	NAA	
CK	0	0	1.20 ± 0.024 h
1	1.0	0.1	2.15 ± 0.012 g
2	1.0	0.5	2.86 ± 0.041e
3	1.0	1.0	2.51 ± 0.026f
4	1.5	0.1	2.91 ± 0.015de
5	1.5	0.5	3.46 ± 0.055c
6	1.5	1.0	3.02 ± 0.038d
7	2.0	0.1	3.64 ± 0.048b
8	2.0	0.5	4.09 ± 0.055a
9	2.0	1.0	2.85 ± 0.052e

Average ± Standard error, different letters in the table indicate significant differences in the 5% level Same below

Notably, during multiple subcultures of the same stem segment explant, the number of axillary buds per explant increased with successive subcultures. Concurrently, as the number of subcultures advanced, the same stem segments explants (used for successive transfers) gradually thickened, the nodes became significantly swollen, and numerous axillary buds differentiated from these swollen nodal regions (Fig. 3B, C).

### Propagation experiment

**Fig. 4:**
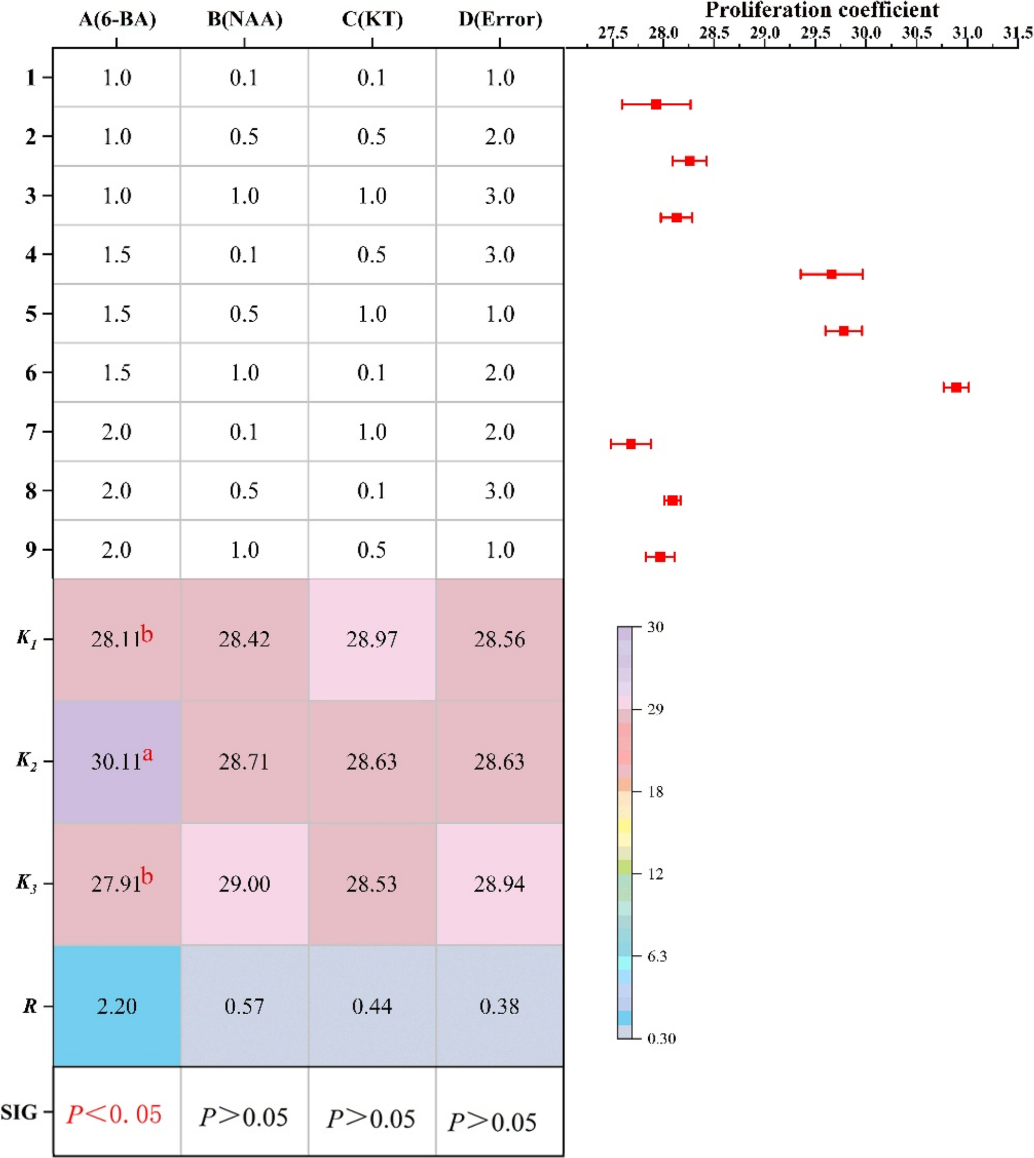
Orthogonal test of L_9_(3^4^) PLB propagation of *A. roxburghii* and its mean heat map-ANOVA-Duncan test results analysis

Since the full factorial combination experiment did not achieve the expected proliferation coefficient, subsequent orthogonal experiments revealed the emergence of numerous green bud primordia at stem nodes close to or inserted into the medium. Repeated verification confirmed that these structures developed from PLBs. Fig. 4 presents the independent results of the L_9_(3^4^) orthogonal experiment on PLB proliferation in *A. roxburghii*, which evaluated the effects of factors A (6-BA), B (NAA), C (KT), and D (error term, for error analysis) on the PLB proliferation coefficient across three levels.

Range (*R*) analysis indicated that the order of influence of each factor on PLB proliferation was as follows: 6-BA (*R* = 2.20) > NAA (*R* = 0.57) > KT (*R* = 0.44) > error term (*R* = 0.38), reflecting the dominant role of cytokinins (primarily 6-BA) in regulating PLB proliferation. Analysis of variance (ANOVA) showed that the effect of 6-BA was significant (*P* < 0.05), while NAA, KT, and the error term did not reach statistical significance (*P* > 0.05). Duncan’s multiple comparison test revealed that the PLB proliferation coefficient induced by Factor A (6-BA) at level 2 (1.5 mg/L 6-BA) was significantly higher than that at level 1 (1.0 mg/L 6-BA) and level 3 (2.0 mg/L 6-BA). By comparing the mean values (*K*) of each factor level, the optimal hormone combination for promoting PLB proliferation was determined to be A_2_B_3_C_1_, i.e., 1.5 mg/L 6-BA, 1.0 mg/L NAA, and 0.1 mg/L KT. This combination yielded the highest PLB proliferation coefficient, and subsequent repeated experiments were conducted to verify its stability.

**Fig. 5:**
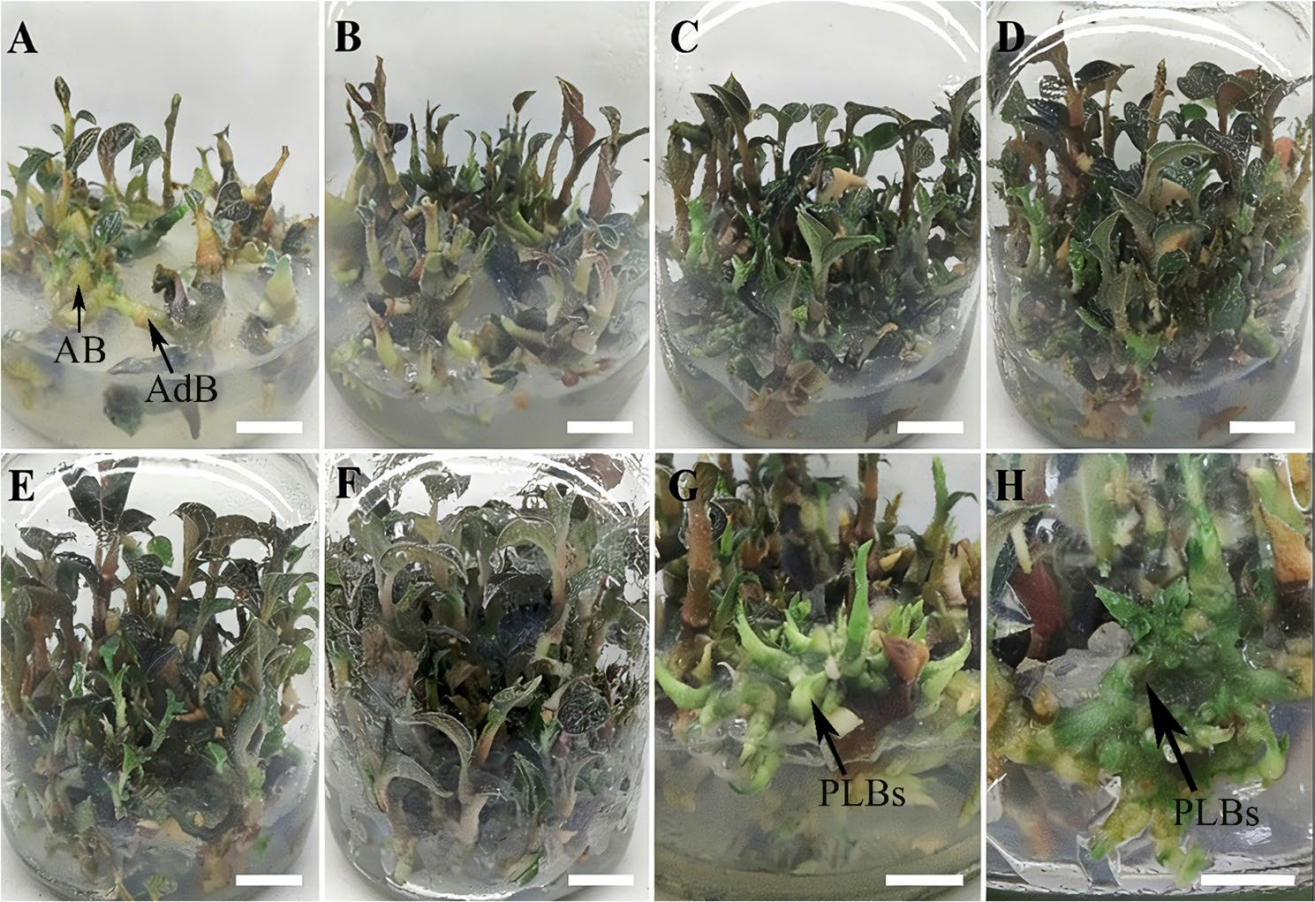
PLB proliferation experiment results of *A. roxburghii* under the optimal PGR combination (1.5 mg/L 6-BA + 1.0 mg/L NAA + 0.1 mg/L KT) **A** After 20 d of culture, the stem nodes expanded, axillary buds and adventitious buds were found on the nodes; **B** After 50 d culture, axillary buds were elongated and PLBs appeared on the basal nodes; **C** After 80 d culture, PLBs increased and some PLBs transformed into new buds; **D** After 110 d culture, the old buds continued to grow and the PLBs continued to differentiate into new buds; **E** After 130 d culture, the growth of old buds was inhibited and new buds continued to grow; **F** After 160 d culture, the regenerated buds filled the whole tissue culture bottle; **G**,** H** PLB clumps produced at nodes after 80 d of culture. Note: AB: axillary buds; AdB: adventitious buds; PLBs: protocorm-like bodies. (Scale bar = 2.0 cm)

Under the optimal hormone combination (A_2_B_3_C_1_:1.5 mg/L 6-BA + 1.0 mg/L NAA + 0.1 mg/L KT), axillary buds began to emerge 20 d after inoculation, with visibly swollen bud bases (Fig. 5A). At 50 d after inoculation, the buds elongated rapidly, the explants further thickened, internode spacing shortened, and PLBs began to form at the basal nodes (Fig. 5B). At 80 d after inoculation, the buds continued to grow, leaves gradually expanded, stems elongated significantly, the number of PLBs increased markedly, and some PLBs differentiated into new shoots (Fig. 5C). At 110 d after inoculation, axillary buds grew vigorously, and PLBs continued to develop and differentiate into complete plantlets (Fig. 5D). At 130 d after inoculation, new shoots continued to grow, while the growth of older buds slowed, though PLBs retained their differentiation capacity (Fig. 5E). At 160 d after inoculation, PLB differentiation was largely complete, and the culture bottles (used for inoculation) were filled with plantlets (Fig. 5F). Compared to the axillary bud regeneration pathway, the formation and differentiation of PLBs were identified as the key factors contributing to the high proliferation coefficient in *A. roxburghii*, with the proliferation coefficient exceeding 30.0 at this stage (Fig. 5G, H).

**Fig. 6:**
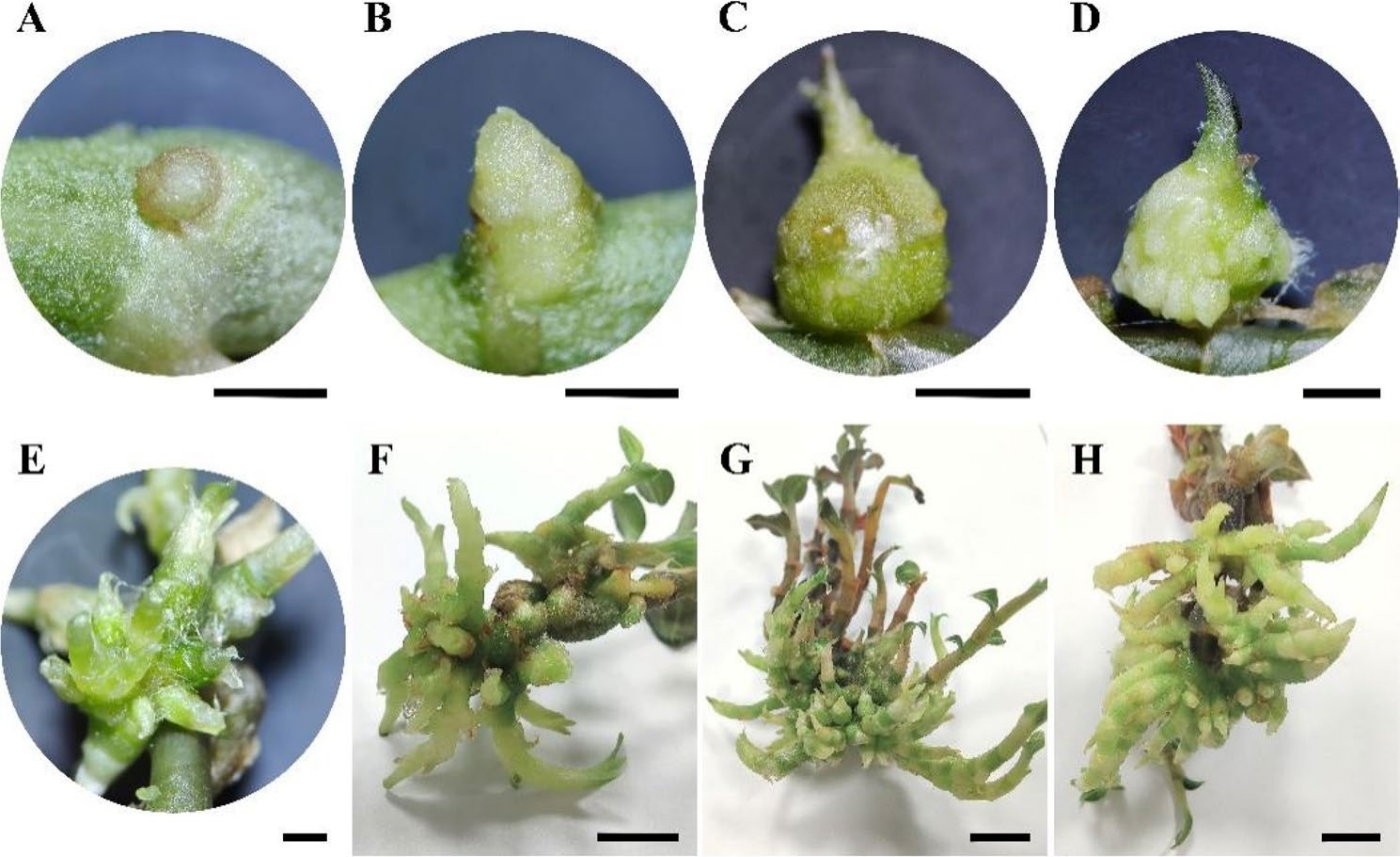
PLB morphogenesis process of *A. roxburghii* under the optimal PGR combination (1.5 mg/L 6-BA + 1.0 mg/L NAA + 0.1 mg/L KT). **A** After 1 0 d of culture, bud primordia appeared on the nodes; **B** After 20 d of culture, the bud primordia continued to grow; **C** After 30 d of culture, the axillary buds swelled in volume and the node region swelled and protruded obviously; **D** After 40 d of culture, protrusions and white villi appeared on the surface of axillary buds; **E** PLBs appeared on axillary bud nodes after 60 d of culture; **F** Adventitious bud clusters were produced after 80 d of culture; **G**,** H** After 100 d of culture, the adventitious buds continued to grow, and bud primordia were formed on the basal nodes of regenerated buds. (Scale bar: **A**-**E**: 20 mm, **F**-**H**: 2.0 cm)

A systematic observation was conducted on the formation process of PLBs in *A. roxburghii*. under the optimal hormone combination (A_2_B_3_C_1_:1.5 mg/L 6-BA + 1.0 mg/L NAA + 0.1 mg/L KT). At 10 d after culturing the explants, yellowish-white bud primordia appeared at the stem nodes (Fig. 6A). At 20 d after culturing, the bud primordia continued to enlarge (Fig. 6B). At 30 d after culturing, the base of the axillary buds significantly swelled, and protruding structures formed at the nodes (Fig. 6C). At 40 d after culturing, yellowish-white tubercular protrusions emerged on the surface of the axillary buds (Fig. 6D). At 60 d after culturing, the nodal regions at the base of the axillary buds further expanded, forming multiple bud primordia that aggregated into clusters of PLBs (Fig. 6E). At 80 d after culturing, the PLB clusters gradually differentiated into multiple adventitious buds (Fig. 6F). At 100 d after culturing, the nodal regions at the base of the axillary buds continued to swell, producing a large number of clustered regenerative shoots with extremely shortened internodes, and new rounds of bud primordia could be observed at the basal nodes (Fig. 6G, H).

It is noteworthy that older stem segments subjected to more than three subcultures formed a mass of swollen meristematic tissue at the basal nodes, with a PLB induction rate approaching 100%. In contrast, stem segments explants with fewer than three subcultures exhibited variable responses. Additionally, after five generations of subculturing, the original linear morphology of the older stem segments explants gradually disappeared, as the stem tissue was replaced by massive PLB clusters formed extensively.

### Results of flow cytometry analysis

**Fig. 7:**
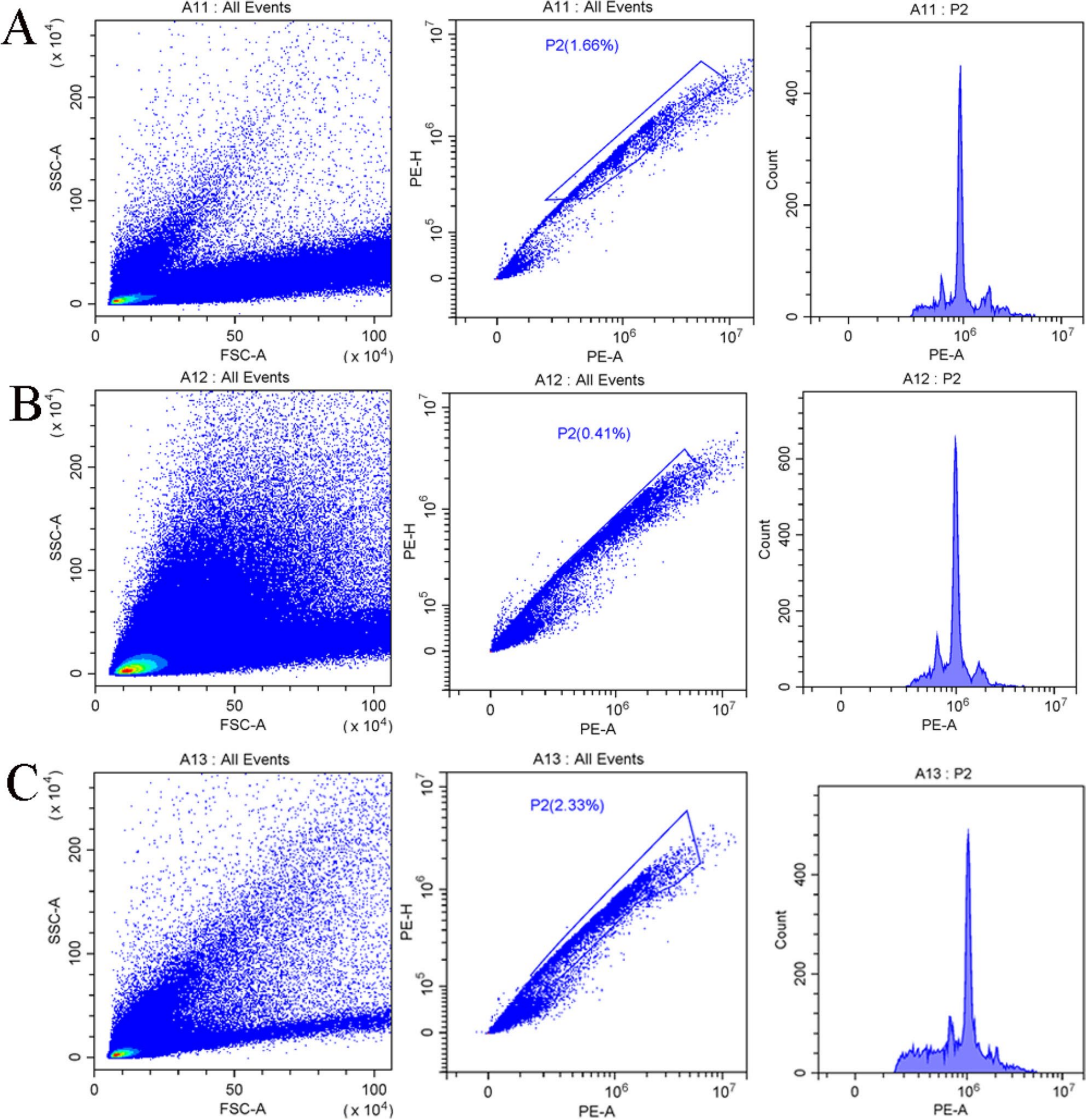
Flow cytometry analysis results of *A. roxburghii* at different culture stages. **A** A11 (primary cultured seedlings); **B** A12 (PLB proliferation stage seedlings); **C** A13 (rooting cultured seedlings). FSC-A (forward scattering light), SSC-A (side scattering light), PE-A (fluorescence signal)

Flow cytometry analysis was performed to detect the ploidy of regenerated materials of *A. roxburghii* at different culture stages, and no ploidy variation was detected, confirming the good genetic stability of the regenerated plants (Fig. 7). The detailed detection results showed that: In sample A11 (primary cultured seedlings), the target cell population P2 accounted for 1.66%. Its FSC-A values were concentrated in the range of 0-100 × 10⁴, the SSC-A value was approximately 200, the PE-A signal was distributed in the 0–10⁷ interval, the cell size uniformity was good, and the internal structure granularity was stable (Fig. 7A). In sample A12 (PLB proliferation stage seedlings), the proportion of the P2 population decreased to 0.41%. The FSC-A values were also concentrated in the range of 0-100 × 10⁴, and the SSC-A value remained around 200. However, the distribution of PE-A signal intensity in the 0–10⁷ interval was more concentrated, with a counting peak reaching 600, suggesting a slight change in cell proliferation activity at this stage (Fig. 7B). In sample A13 (rooting cultured seedlings), the proportion of the P2 population increased to 2.33%(Fig. 7C). The FSC-A and SSC-A parameters were basically consistent with those of A11 and A12. The PE-A signal showed a uniform distribution in the 0–10⁷ interval, with a counting peak of 400, and the characteristics of the cell population were stable. Overall, there were no significant differences in the flow cytometry parameters among the three samples, and no abnormal ploidy cell peaks appeared. Combined with the regeneration characteristics mentioned earlier, where PLBs occur directly without a callus stage, it further confirms that the “stem node-induced PLB regeneration system” established in this study can ensure the genetic stability of regenerated *A. roxburghii* plants, providing cytological support for the subsequent large-scale production of seedlings and the consistency of genetic background.

### Rooting culture and transplantation

The rooting results are shown in Table 3. Except for the blank control group (MS medium without NAA), the rooting rate reached 100% in all NAA-treated groups, indicating that NAA significantly promoted the formation of adventitious roots in tube plantlets of *A. roxburghii*. The average number of adventitious roots peaked at an NAA concentration of 0.5 mg/L; beyond this concentration, the average number of adventitious roots gradually decreased, suggesting that lower NAA concentrations (0.1–0.5 mg/L in this experiment) are more conducive to root formation in *A. roxburghii*. Although the average number of adventitious roots at 0.1 mg/L NAA was lower than that at 1 mg/L, the adventitious roots at 1 mg/L NAA were thin and weak, easy to brown, and with low root activity; while the adventitious roots at 0.1–0.5 mg/L NAA were robust, with rich root hairs and high root activity. Moreover, 0.5 mg/L NAA treatment achieved 100% rooting rate and the highest number of robust adventitious roots at the same time. Therefore, low concentration of NAA (0.1–0.5 mg/L) is more suitable for high-quality rooting culture of *A. roxburghii*, rather than simply pursuing the number of roots.

**Table 3:** Effect of different NAA concentrations on rooting of *A. roxburghii*

NO.	NAA (mg∙L^− 1^)	The average number of adventitious roots	Growth conditions
1	0	0.19 ± 0.075d	There were basically no roots
2	0.1	1.12 ± 0.121c	Roots long and thin more sparsely, low root activity
3	0.5	2.87 ± 0.087a	Roots robust developed, covered with white hairs, high root activity
4	1.0	2.25 ± 0.117b	The root system is more developed, the number of roots decreased, and roots were easy to brown

**Fig. 8:**
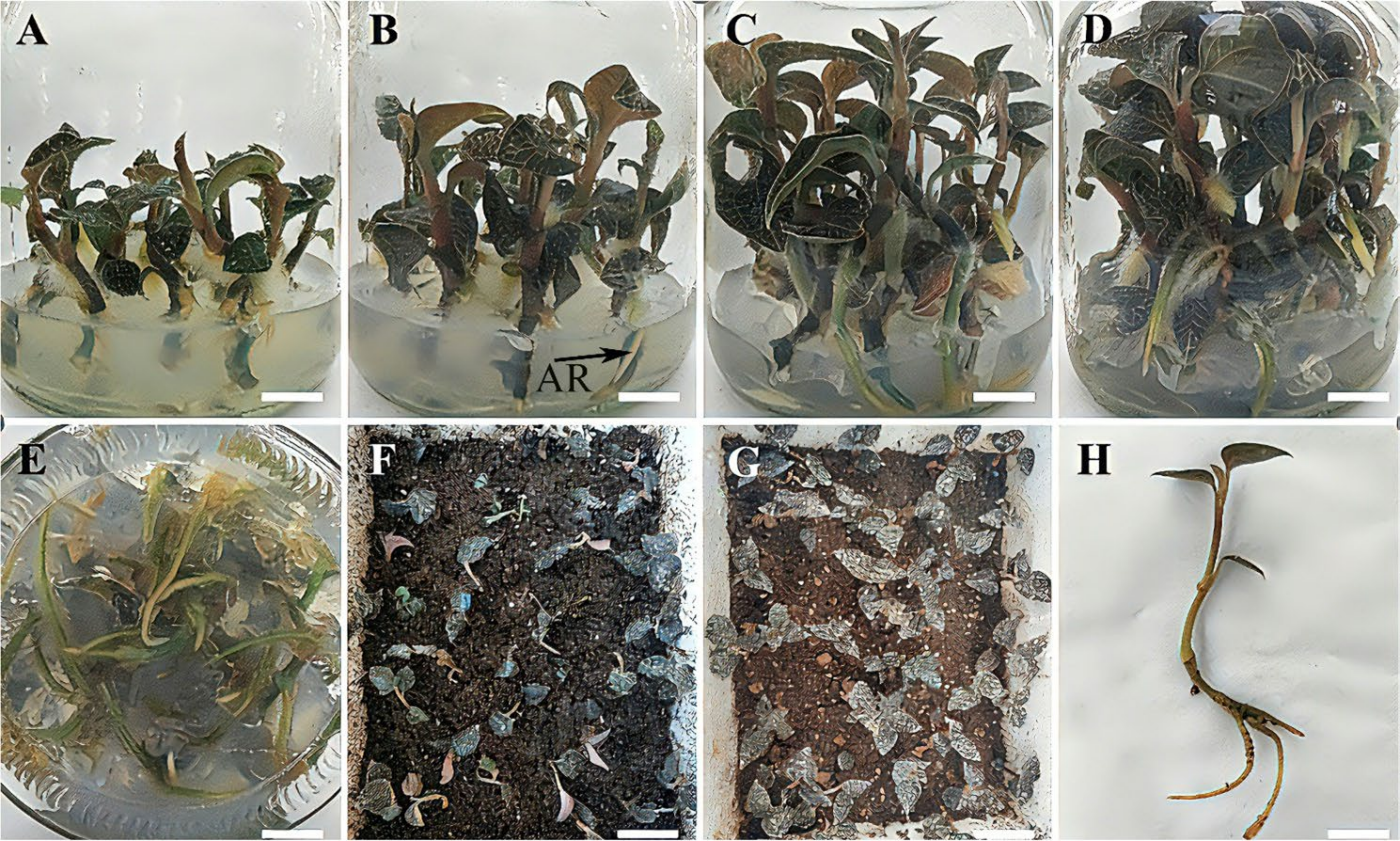
Rooting culture and transplanting results of *A. roxburghii* in MS medium with 0.5 mg·L⁻¹ NAA. **A** After 30 d of culture, root primordia and white root tips appeared; **B** After 60 d of culture, the plants grew and the adventitious roots elongated; **C** After 90 d of culture, the plants grew well and the number of adventitious roots increased; **D**,** E** After 120 d of culture, the plants were flourishing and the adventitious roots were developed; **F**. Transplanted plantlets in foam boxes; **G** Rooted plantlets grew well after transplanting for 60 ds; **H** Robust root system after 60 d of transplanting growth. Note: AR: Adventitious roots. (Scale bar = 2.0 cm)

In the treatment with 0.5 mg/L NAA, white filamentous adventitious root primordia formed at the nodes of the stem segment explants after 30 d of culture, and tiny root cap structures were visible at the tip of the root primordia, accompanied by short white root tips emerging (Fig. 8A). After 60 d of culture, the plantlets grew normally, with robust white adventitious roots observed (Fig. 8B). By 90 d of culture, the plantlets continued to grow, producing numerous adventitious roots that transitioned from white to yellowish-green and exhibited obvious geotropic growth (Fig. 8C). After 120 d of culture, the plantlets thrived, with the culture bottles filled with an extensive and sturdy root system—providing a solid foundation for subsequent acclimatization and transplantation (Fig. 8D, E). The rooted plantlets were transplanted into foam boxes for acclimatization (Fig. 8F). After 60 d of transplantation, the stems elongated, leaves expanded, and the plants exhibited healthy growth, with a survival rate exceeding 95% (Fig. 8G, H). Low concentration of NAA (0.1–0.5 mg/L) specifically induces the differentiation of adventitious roots and adventitious buds at the nodal regions of stem segment explants of *A. roxburghii*, and no root/bud differentiation was observed at the shoot tip regions.

## Discussion

### Propagation modes and protocorm-like body formation in *A. roxburghii* (Wall.) Lindl.

Tissue culture propagation techniques for orchids have been practiced for over a century, and artificial clonal propagation systems have been established for many endangered species within this family. Regeneration pathways include in vitro shoot proliferation, direct adventitious bud formation, seed-derived protocorms, as well as PLBs or calli induced from explants such as protocorms, stem nodes, shoot tips, leaves, root tips, or tubers [16, 19, 20]. However, orchid explants rarely form callus under in vitro conditions, and the pathway of inducing regenerative shoots via callus is more challenging compared to other methods [16, 20]. Against this background, this study primarily focuses on two regeneration pathways for *A. roxburghii*: axillary buds and PLBs induced from stem segments explants.

In the full factorial combination experiment, each stem segment explants produced 1–2 axillary buds. As the number of subcultures increased, the stem segments explants gradually thickened, the nodes swelled, and multiple axillary buds formed. This phenomenon in *A. roxburghii* may originate from the presence of axillary meristems in the leaf axils of its leaf primordia, which have the potential to differentiate into new vegetative structures, inflorescence meristems, or vegetative/reproductive branches—resembling characteristics observed in certain horticultural plants [21]. Axillary meristems may derive from shoot apical meristems or arise via dedifferentiation of cells in the leaf axils; however, both sources of axillary meristems are inhibited by apical dominance [22]. When stem segments explants of *A. roxburghii* are freed from apical dominance (via stem segment excision) and induced by cytokinins (e.g., 6-BA) used in this experiment, the axillary meristems in leaf axils can differentiate into axillary bud primordia. With increasing subculture cycles, the axillary meristems within the same stem segment explants continue to differentiate, producing more axillary buds.

A previous study established a “PLB Induction–Proliferation–Regeneration” (IPR-PLB) system for *A. roxburghii*; however, the secondary PLB proliferation rate (defined as the ratio of final PLB number/biomass to initial number/biomass) only reached 400% (4-fold) in number and 350% (3.5-fold) in biomass [14]. Moreover, the site of PLB formation and its cellular origin were not clearly identified, limiting its applicability for large-scale seedling production and theoretical research. In contrast, the PLB proliferation coefficient achieved in this study was more than 30 times, which was much higher than the 4 times proliferation efficiency reported by Wang et al. [14]. Through innovative hormone combinations and temporal morphological tracking, the present study achieved a dual breakthrough in both proliferation efficiency and the understanding of PLB formation mechanisms. In the orthogonal experiment of this study, the basal nodes of regenerated shoots became significantly swollen and directly formed PLBs. The occurrence of PLBs not only markedly improved the efficiency of asexual propagation, but also largely bypassed the callus phase, thereby reducing the possibility of variation in regenerated plantlets [23]. Meristem cells are undifferentiated cells with stable chromosome ploidy. Direct induction of PLBs from stem node meristems without the dedifferentiation process of callus avoids somatic clonal variations such as chromosome replication errors and karyotype variation caused by rapid cell division during dedifferentiation; flow cytometry results showed no ploidy variation in regenerated materials at different culture stages, which directly confirmed the genetic stability advantage of this pathway, while the somatic variation rate of callus induction pathway can reach more than 10% due to the randomness of cell dedifferentiation [24]. PLBs derived directly from meristematic tissues exhibit higher genetic stability than those formed via callus [24]. In this study, the PLBs originated from malformed axillary buds on one side of the stem nodes. These axillary buds exhibited extremely shortened internodes and developed yellowish-white spherical granular protrusions on the surface, which further developed into PLB clusters over time. Throughout this process, no callus involvement was observed, and the PLBs formed directly at the nodal regions. This differs from findings in *Anthurium andreanum* cv. CanCan and *Dendrobium chrysotoxum* Lindl., in which PLBs are typically induced directly or indirectly from shoot tip explants [20, 23, 25]. Stereomicroscopic observations revealed that these PLB clusters originated from the basal nodal regions of the axillary buds, and vascular bundle connections were clearly visible at the base of young shoots derived from the PLBs. Combined with the results of the orthogonal experiment, it is preliminarily hypothesized that 6-BA promotes PLB formation by activating the division of vascular cambium cells, further supporting the possibility that PLBs originate from the vascular tissue at the basal nodes of axillary buds. This morphological and experimental evidence compensates for the research gap left by Wang et al. who only demonstrated the macroscopic morphology of PLBs without detailing their vascular origins [14].

PLBs can be induced from a variety of explant types through diverse developmental pathways. Multiple cell types, including epidermal cells, vascular bundle cells, subepidermal cells, and individual cells within callus tissues, possess the capacity to differentiate into PLBs [9, 26]. Unlike the axillary bud formation observed in the full factorial combination experiment, PLB formation in the orthogonal experiment of this study was characterized by the initial development of only a single axillary bud per stem node, exhibiting a phyllotactic arrangement analogous to the alternate leaf pattern of *A. roxburghii*. This axillary bud demonstrated dual developmental potential: it could undergo normal growth or produce numerous PLBs at its basal nodes. As the base of the axillary bud thickened and expanded, PLBs formed clustered aggregates around the nodal region, with each PLB maintaining vascular connections to the original explant [27].

The distinctive swollen axillary nodes observed in this study fundamentally represent the outcome of sustained differentiation within axillary meristematic tissues. Morphologically, these structures resemble the root nodules of woody plants, constituting compact and relatively autonomous cellular masses. Their internal organization includes meristematic cells, plastid-rich parenchyma cells, vascular elements, as well as differentiated epidermal, cortical, and vascular tissues [28]. Under appropriate cultural conditions, such nodules can directly undergo organogenesis to form shoots and roots, ultimately developing into complete plantlets [28, 29]. Similarly, direct reciprocal conversion between PLBs and rhizomes has been documented in the genus *Cymbidium* [30].

This investigation reveals a sequential developmental transition from axillary buds to PLBs in stem segments explants of *A. roxburghii*. Individual explants progressively activated these two distinct regenerative pathways (axillary bud formation and PLB formation) with increasing subculture cycles. Furthermore, PLB formation demonstrated specific positional preference (occurring at nodes distal to the apex) and asynchronous development. These findings not only corroborate the regulatory influence of apical dominance [22] but also propose a novel regeneration pathway in *A. roxburghii* that operates independently of callus formation. This mechanism shows parallels to the non-embryogenic developmental pattern reported in *Phalaenopsis amabilis* (L.) Blume [31], thereby contributing new insights into the regenerative diversity among orchid species.

### Synergistic effects of PGRs in the micropropagation of *A. roxburghii* (Wall.) Lindl.

PGRs play a crucial role in regulating plant cellular totipotency and regenerative capacity, with their types and concentrations significantly influencing cell dedifferentiation, proliferation, growth, and morphogenesis [32]. The PGR screening experiments in this study not only demonstrated distinct functional differences among hormone categories, but also revealed their synergistic effects (e.g., 6-BA and NAA promoting PLB proliferation): cytokinins (e.g., 6-BA, KT used in this study) primarily promoted adventitious bud formation, while auxins (e.g., NAA, 2,4-D used in this study) facilitated adventitious root development. The differentiation capacity of meristematic tissues is co-regulated by endogenous and exogenous signals, with axillary meristems particularly influenced by inhibitory endogenous signals from shoot apical meristems [33, 34]. Consistent with this, the shoot tips of *A. roxburghii* in this study rarely produced adventitious buds under any PGR treatment. Meanwhile, low concentrations of NAA (0.1–1.0 mg/L) not only effectively induced adventitious roots but also promoted bud formation, underscoring the dual roles of low concentrations of auxins in stimulating vascular bundle differentiation and participating in the morphogenesis of both shoots and roots [35].

The application of 6-BA alone induced adventitious bud formation, but the resulting adventitious buds consistently exhibited malformed morphologies (e.g., shortened internodes, distorted leaves) and failed to develop into normal plantlets. In contrast, when NAA and KT were added in combination with 6-BA (the basic cytokinin in this study), both axillary buds and PLBs formed simultaneously at the stem nodes, indicating that synergistic interactions of multiple PGRs (6-BA, NAA, KT) are more conducive to efficient propagation of *A. roxburghii*. The growth mechanism of axillary buds depends on the endogenous cytokinin-to-auxin ratio, with lower concentrations of auxin generally favoring the activity of axillary meristems [22]. In the full factorial combination experiment, the combination of high concentrations of 6-BA and low concentrations of NAA resulted in an axillary bud induction rate of up to 100% in stem segments explants. With successive subcultures, continuous stimulation by exogenous hormones may alter endogenous feedback inhibition mechanisms, promoting accelerated division of axillary meristematic cells. When the rate of cell division exceeds that of cell elongation, nodal swelling occurs and more axillary buds are produced. Previous studies have reported that excessive suppression of auxin signaling in leaf axils (e.g., by low exogenous auxin concentrations) can lead to a significant increase in the number of axillary buds [36].

The combination of 6-BA and NAA is commonly used in proliferation cultures of Orchid species, while 6-BA together with TDZ is widely employed for PLB induction [10, 37]. In *A. roxburghii*, the combination of 6-BA and NAA alone (at the concentrations tested in this study) did not induce PLB formation. However, after the addition of a low concentration of KT (0.1 mg/L), malformed axillary buds appeared on the stem nodes and further differentiated into PLBs. This regulatory mechanism fundamentally differs from the PLB induction system reported by Wang et al. which relied solely on 6-BA, NAA, ZT (zeatin), and 2,4-D without incorporating KT, and achieved only a 4-fold proliferation efficiency [14]. In contrast, the present study utilized KT for its role in “synergistically releasing apical dominance” (rather than directly promoting division), forming a tripartite regulatory circuit with 6-BA and NAA: 6-BA activates vascular cell division, KT putatively inhibits the expression of auxin transporters (e.g., PIN1) to release axillary bud suppression, and NAA balances the direction of cell differentiation, ultimately achieving a 30-fold proliferation rate. As an adenine-type cytokinin, KT exhibits strong regulatory capabilities in cell division and bud differentiation [38]. PLB formation in most orchids depends on the coordination of cytokinins and auxins, though the specific concentrations, combinations, and ratios vary significantly among species [39].

Similar to the PLB induction systems established in other recalcitrant orchid species, the success of our protocol relies heavily on the precise synergy of cytokinins [40, 41]. In Paphiopedilum orchids, secondary PLB formation from primary PLBs was also found to be highly dependent on cytokinin type and concentration, with kinetin proving superior to 6-BA at higher concentrations [40]. This aligns with our finding that KT is an essential component for breaking the axillary bud meristem fate and initiating PLB development in *A. roxburghii.* Furthermore, the direct induction pathway observed in our study (from stem nodes) shares striking similarities with the leaf segment-based direct PLB induction reported in Vanda coerulea hybrids [41]. In that study, TDZ was used to achieve direct PLB induction at a high frequency (94.8%), bypassing the callus phase entirely [41]. Although our study used KT instead of TDZ, the core principle of using a specific cytokinin trigger to unlock direct somatic embryogenesis (PLB formation) is consistent across these divergent orchid genera. This cross-species conservation of regulatory logic further validates the robustness of our established protocol.

It should be noted that the present study focused on PLB proliferation efficiency and hormonal regulatory mechanisms, and did not evaluate the content of pharmacologically active compounds in the highly proliferating PLBs. This is a limitation of this study Wang et al. [14], using HPLC-ELSD technology, demonstrated that the kinsenoside (the core bioactive compound of medicinal *A. roxburghii*, commonly referred to as “Jinxianlian glycoside” in Chinese) content in PLBs of *A. roxburghii* could reach a peak of 34.27 mg/g FW (close to the 38.68 mg/g FW found in whole plants), and that astragalin accumulated only during the first two weeks of PLB culture. Similar to the findings in *Dendrobium Sabin Blue*, where PLBs were successfully utilized as a novel source for the in vitro production of specialized metabolites like dendrobine and anthocyanin [42], the PLBs in this study also showed strong proliferation ability and genetic stability, which laid a foundation for their potential application in the production of active compounds.

Subsequent studies should first verify the repeatability of this HPLC-ELSD method in our culture system, then detect the content of kinsenoside and flavonoids (such as isoquercitrin and astragalin) at different proliferation stages in our system (e.g., the 20-d axillary bud swelling stage and the 60-d PLB cluster stage after inoculation). This would verify whether “high proliferation is accompanied by active compound degradation” and, combined with the developmental timeline of PLBs, identify the optimal extraction time. Ensuring that this system possesses both “quantitative advantage” and “medicinal value” will provide data support for the integrated industrialization of *A. roxburghii*, from seedling propagation to active compound extraction. In addition, subsequent studies will further clarify the cellular origin and embryogenic process of PLBs in *A. roxburghii* through histological methods such as paraffin sectioning and scanning electron microscopy, so as to further reveal the molecular mechanism of its direct induction. 

### The application value and industrialization potential of *A. roxburghii* (Wall.) Lindl. in this regeneration system

The “efficient regeneration system of PLBs induced from stem nodes” established in this study not only solves the problem of low in vitro propagation efficiency of *A. roxburghii* at the technical level, but also provides stable seedling support for its wild-simulated cultivation under forests in practical applications. It has both ecological protection and economic value.

In terms of seedling production capacity, this system has core advantages such as a proliferation coefficient exceeding 30.0, a rooting rate of 100%, and a transplant survival rate exceeding 95%. The efficiency of seedling propagation is significantly better than that of traditional tiller propagation and conventional tissue culture systems. Starting with 20 healthy stem node explants, after the first round of 160-d proliferation culture, 20 × 30 = 600 effective regenerated buds can be obtained; after the second round of 160-d proliferation culture, 600 × 30 = 18,000 effective regenerated buds can be obtained; followed by 120-d rooting culture and acclimatization and transplantation, ultimately up to 18,000 robust seedlings can be obtained in one year. This achieves exponential growth in the number of seedlings, effectively solving the problem of seedling shortage in understory wild-simulated cultivation.

From the perspective of matching the scale of understory cultivation, combined with the planting density of 500 plants/m2 for understory wild-simulated cultivation [43] the understory planting area is 18,000 ÷ 500 = 36 m2. This scale is suitable for the production needs of small and medium-sized growers. At the same time, the planting area can be further expanded through multiple rounds of propagation. Moreover, after domestication, the seedlings can quickly adapt to the wild-simulated environment under the forest with “70%-80% canopy density and a temperature of 20–30 ℃ “. The survival rate and growth stability after transplantation are significantly higher than those of unacclimated tissue culture seedlings, laying a foundation for *A. roxburghii* to reach the “special grade 1” quality.

In summary, this system provides key support for understory wild-simulated cultivation through efficient seedling propagation. It not only alleviates the pressure of endangerment on wild *A. roxburghii* resources but also offers an integrated “technology-capacity " solution for the industrialization of medicinal plants. It has significant practical significance for promoting the sustainable utilization of *A. roxburghii* and the development of understory economies in mountainous areas.

## Conclusion

This study established a highly efficient in vitro regeneration system for *A. roxburghii* (Wall.) Lindl. via direct PLBs induction from stem nodes, bypassing callus formation. The optimal hormone combination (1.5 mg/L 6-BA, 1.0 mg/L NAA, and 0.1 mg/L KT) achieved a remarkable proliferation coefficient exceeding 30.0, while 0.5 mg/L NAA added to full-strength MS medium yielded a 100% rooting rate. Plantlets acclimatized successfully with > 95% survival, and flow cytometry analysis confirmed the genetic stability of regenerated plants. This PLB-mediated pathway demonstrates significant potential for rapid, large-scale propagation of this endangered medicinal orchid, offering a robust solution for conservation and commercial production.

Future studies should focus on three aspects: (1) Validate the content of key bioactive compounds (e.g., kinsenoside) in PLBs and regenerated plants using HPLC to ensure the medicinal value of the propagation system; (2) Conduct large-scale field cultivation trials to verify the adaptability and yield stability of seedlings derived from this system; (3) Explore the molecular mechanism of PLB direct induction by analyzing the expression patterns of key genes involved in cytokinin signaling and embryogenesis.

## Data Availability

Data will be made available upon reasonable request.

## Abbreviations

2,4-D: 2,4-dichlorophenoxyacetic acid
6-BA: 6-benzylaminopurine
ANOVA: Analysis of variance
CV%: Coefficient of variation
HPLC: High-performance liquid chromatography
IBA: Indole-3-butyric acid
KT: Kinetin (6-furfurylaminopurine)
MS: Murashige and Skoog
NAA: 1-naphthaleneacetic acid
PLBs: Protocorm-like bodies
PGRs: Plant growth regulators
PI: Propidium iodide
SE: Standard error
